# Bone transport combined with sequential nailing technique for the management of large segmental bone defects after trauma

**DOI:** 10.3389/fsurg.2024.1302325

**Published:** 2024-01-18

**Authors:** Qian Wang, Teng Ma, Zhong Li, Kun Zhang, Qiang Huang

**Affiliations:** Department of Orthopedics, Hong Hui Hospital, Xi’an Jiaotong University, Xi’an, Shaanxi, China

**Keywords:** bone transport, segmental bone defects, intramedullary nail, bone graft, trauma

## Abstract

**Background:**

Bone transport technique is widely used for the management of large segmental bone defects. However, several reasons may prevent its successful completion, such as poor osteogenesis, docking site nonunion, severe chronic pain and psychological problems. We used sequential nailing technique to solve these problems. The objective of this study was to analyze the clinical effects of our modified technique for the management of large segmental bone defects after trauma.

**Methods:**

Twenty-three patients using bone transport combined with sequential nailing technique in our institution from June 2011 to June 2020 were included and analyzed retrospectively. There were 15 males and eight females. The age ranged from 19 to 64 years. There were eight cases suffering from basic medical diseases. The initial injury was open in 14 patients. Seven cases encountered femoral defects and 16 for tibia. The main reasons for sequential nailing technique were docking site nonunion (nine cases), poor osteogenesis (five cases), severe chronic pain (five cases) and psychological problems (four cases). The residual bone defects after removing the external fixator, operation plans, complications and follow-up time were recorded. Bone defect healing was evaluated by Paley score.

**Results:**

The mean residual bone defects were (2.9 ± 1.9) cm. The mean time in external fixator was (9.5 ± 3.4) months. The average follow-up time was (23 ± 3) months. With respect to complications, two cases suffered from nonunion again and were treated by bone graft with augmented plate fixation. No infection recurrence was found in these cases. The excellent and good rate of bone defect healing was 91.3%.

**Conclusion:**

Bone transport combined with sequential nailing technique could shorten the external fixation time, overcome the inconvenience of the external frame to patients, eliminate chronic pain and be easy for patients to accept. Patients using this modified technique achieved high satisfaction.

## Background

The management of large segmental bone defects after trauma is difficult. These patients are often accompanied with large skin and soft tissue defects, infection, etc. (1). If the treatment is not timely, it may even lead to amputation. Therefore, these patients face great physical, psychological and economic burden.

Bone transport technique is an effective treatment method for patients with large segmental bone defects (2, 3). It emphasizes the flexible use of tension-stress law, individualized selection of fixation methods, maximum protection of autologous tissues, and the use of randomly combined stable spatial mechanical structure to mobilize the repair potential of the body's own tissues (4, 5). The commonly used fixation methods including Ilizarov annular fixation and Wagner (single-arm) external fixation. The transport schemes include the uni-focal, bifocal, trifocal transport, etc. (6, 7). Although this technique has saved lots of limbs on the verge of amputation, it still has some obvious shortcomings.

One of the major shortcomings is the long-lasting consolidation period, which may cause lots of complications, such as pin-tract infection, axial deviation, joint stiffness, etc. (8, 9). When dealing with these complications, it will further prolong the external fixation time, making the external fixation time longer. Wearing an external fixator for a long time brings great inconvenience to patients (10, 11). This makes many patients unable to return to normal life for one or even more years. Some patients may encounter psychological problems and severe chronic pain during bone transport process. These patients strongly request removal of the transport frame. Docking site nonunion is another common complication in using bone transport technique (12). In our previous study, its incidence could be as high as 29% (12/41) (13). The occurrence of this complication increases the number of operations and prolongs the time of external fixation. Some patients ask to remove the external fixator and change the treatment plan when encountering docking site nonunion. In addition, a few patients encounter poor osteogenesis of the new callus during bone transport.

The above factors make it difficult to continue bone transport process. Several extreme patients may ask for termination of treatment and even ask for amputation. The emergence of these situations urges trauma surgeons to change treatment plans at this time. That is, surgeons have to balance therapeutic effects and limb tolerance. We used bone transport combined with sequential nailing technique for the management of large segmental bone defects after trauma. When patients encounter poor osteogenesis, severe chronic pain, docking site nonunion, or obvious psychological problems and strongly require removal of the transport frame, it would be removed in time, and replaced with an intramedullary nail and bone graft. This is a continuous treatment process for patients. The intramedullary nail was used to maintain limb stability until bone defect healing. From June 2011 to June 2020, 23 patients in our institution were treated by this modified technique. These patients achieved good clinical results and high satisfaction. It is reported as follows.

## Materials and methods

### Inclusion criteria

(1) Patients over 18 years; (2) Patients with large segmental bone defects after trauma; (3) Patients who were treated by the bone transport combined with sequential nailing technique; (4) The clinical and imaging data were complete.

### Exclusion criteria

(1) Patients younger than 18 years; (2) Patients suffered from severe medical diseases and were unable to tolerate anesthesia or a surgery; (3) Patients suffered from severe limb damage and received amputation treatment; (4) Patients with incomplete clinical data; (5) Lost patients.

### Preoperative treatment

For patients with bone defects resulting from infection or osteomyelitis, the inflammatory indexes were detected before operation, including blood routine, erythrocyte sedimentation rate (ESR), C-reactive protein (CRP), procalcitonin. The wound secretion was taken for bacteriological culture and drug sensitivity test. The full-length x-ray images of femur or tibia were performed. CT or MRI would be performed if necessary. The bone transport frame was removed before nailing operation. The necrotic tissues in the pin-tracts were scraped off thoroughly. The injured limb was temporarily fixed using a plaster or brace. One week later, when the pin-tracts healed and no abnormality was found in infection-related indicators, sequential nailing would be performed. Very few patients may experience delays in replacing intramedullary nails due to slower pin-tract healing.

### Operation steps

**Figure 1:**
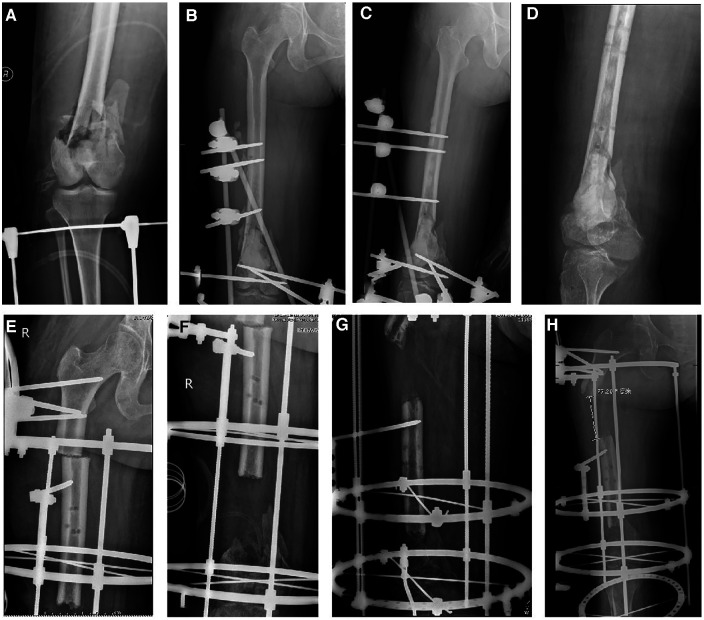
A 52-year-old female suffered from a serious open fracture and caused large segmental femoral defects. (**A**) x-ray images at initial injury; (**B,C**) x-ray images after debridement and filling with bone cement; (**D**) x-ray images after removal of the temporary external fixator; (**E,F**) x-ray images when bone transport frame was installed; (**G,H**) Poor osteogenesis occurred after 15 months of bone transport.

**Figure 2:**
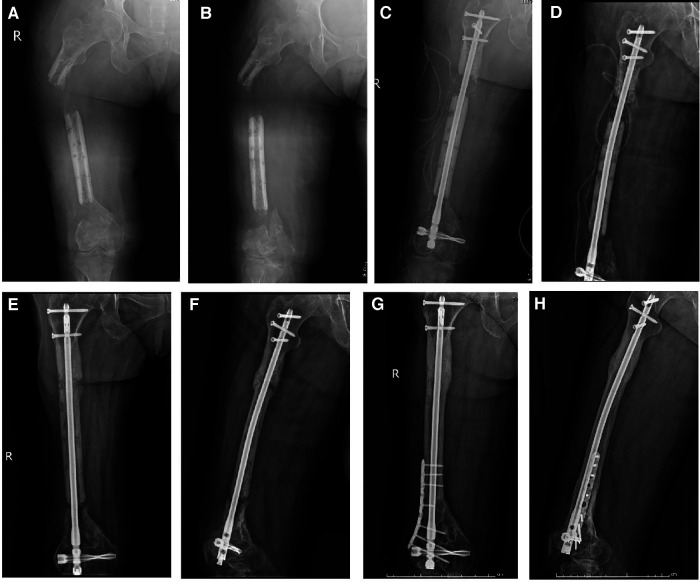
The 52-year-old patient was successfully treated by sequential nailing and bone graft technique. (**A,B**) After removing the bone transport frame, 7 cm of poor osteogenesis was observed; (**C,D**) A retrograde femoral intramedullary nail was inserted and autologous bone graft was performed; (**E,F**) After 9 months, nonunion still existed at the docking site; (**G,H**) After augmented plate fixation and bone graft, the docking site healed well.

**Figure 3:**
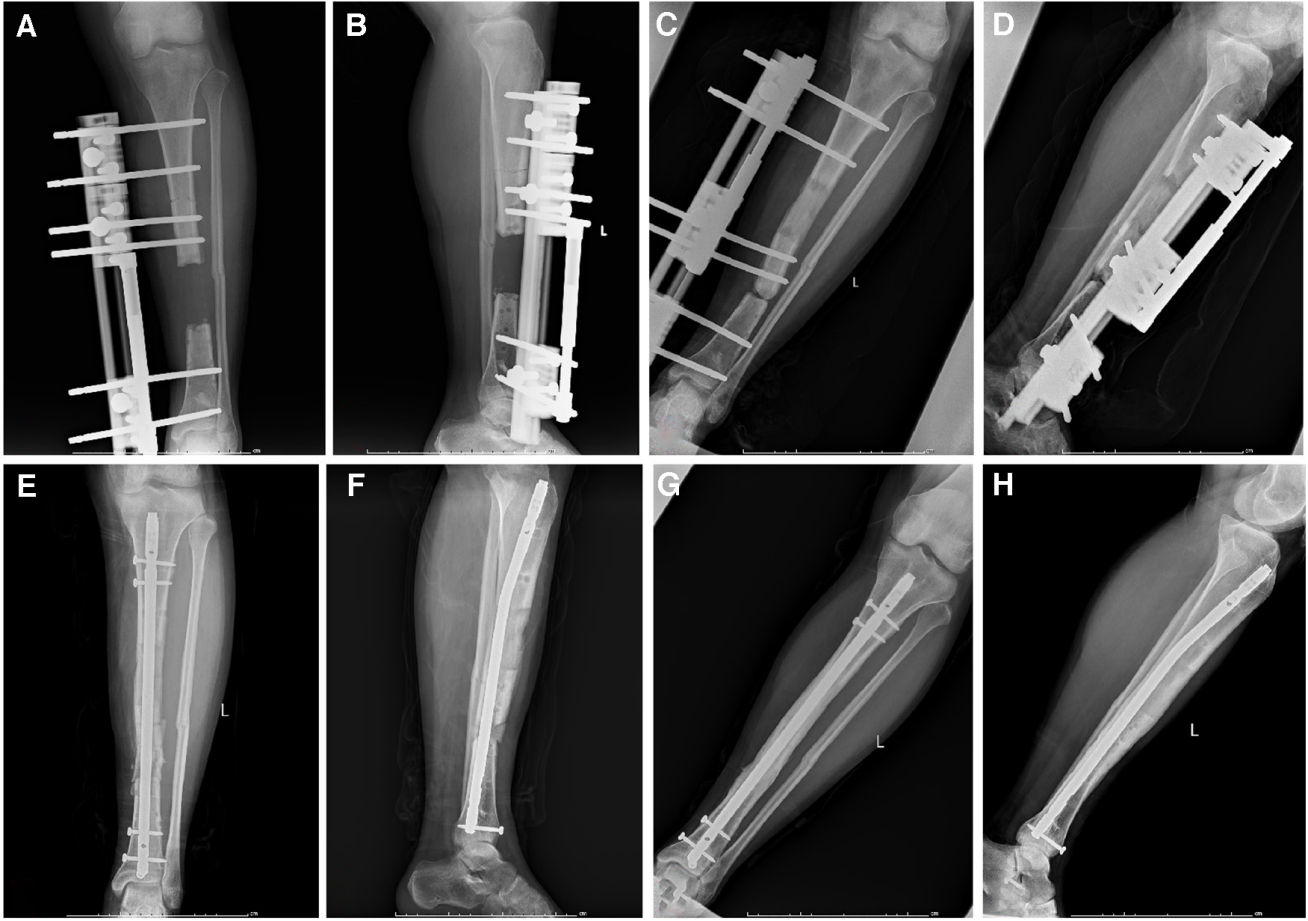
A 26-year-old male suffered from large segmental bone defects of tibia. (**A,B**) After segmental resection, the single-arm frame was installed; (**C,D**) Docking site nonunion occurred when bone transport nearly finished; (**E,F**) After removal of the transport frame, sequential nailing and bone graft was performed; (**G,H**) One year after removing the external frame, the docking site healed well.

The operations were performed by the same senior surgeons. Taking the bone defect site as the center, a lateral longitudinal incision of 8–10 cm was made. The residual bone defects were exposed. Both ends of the bone defects were debrided again. The ischemic sclerotic bones and scar tissues were removed. If there was obvious angular deformity at the distraction segment, wedge osteotomy could be performed. When poor osteogenesis occurred, the scar tissues occupying this space should be removed and the osteogenic channel should be reconstructed. Then, according to the surgical design, an intramedullary nail (IRENE, Tianjin, China) was inserted. The entry point of the nail was opened. The guide wire passed through the distraction segment and entered into the distal medullary cavity. As the medullary cavity of the distraction segment did not form, the cortical bones of this segment were weak. The guide wire should be kept not to penetrate out of the bone cortex. After that, the medullary cavity was expanded with a reamer along the guide wire. Under an image intensifier, the limb length was maintained. The alignment and rotation were adjusted. An intramedullary nail with appropriate length and diameter was selected and inserted into the medullary cavity. The distal and proximal ends were fixed by interlocking screws, respectively. The residual bone defects were measured, and autologous iliac bones were taken for granular bone graft. Finally, a drainage tube was placed and the incisions were closed. Under the image intensifier, it was confirmed that the transplanted bones were sufficient and the position of internal fixation devices was good. Two typical cases are shown in Figures 1–3.

### Postoperative treatment

All patients were treated with anti-infection and anti-coagulation treatment after operation. Antibiotics were adjusted in time according to the drug sensitivity test. Inflammatory indexes were detected regularly. In order to prevent joint stiffness and muscle atrophy, limb function exercises began immediately after operation. The specific weight-bearing time was determined according to the healing of residual bone defects.

### Observation indexes

The residual bone defect length, operation plans, complications and follow-up time were recorded. At the last follow-up, the bone defect healing was evaluated by Paley score (14). The grade of bone defect healing assessed by Paley score is as follows: (1) Bone healing; (2) There is no recurrence of infection; (3) Limb deformity less than 7°; (4) The unequal length of limbs is less than 2.5 cm. Excellent: good bone healing + (2)–(4) indexes are met. Good: good bone healing + (2)–(4) indexes meet two items. Fair: good bone healing + (2)–(4) index meets one item. Poor: bone nonunion or re-fracture + (2)–(4) indexes were not met.

### Statistical analysis

SPSS 24.0 software was used to process data. Measurement data were expressed as mean ± standard deviation by unpaired *t*-test. Count data were analyzed using *χ*2 test. *P *< 0.05 was defined as statistically significant.

## Results

### Demographic information of 23 patients

There were 15 males and eight females using bone transport combined with sequential nailing technique. The age ranged from 19 to 64 years, with a mean age of (41 ± 12) years. There were eight cases suffering from basic medical diseases, including hypertension, coronary heart disease (CHD), diabetes and nephropathy. The initial injury was open in 14 patients. Seven cases encountered femoral defects and 16 for tibia. The main reasons for removing the transport frame were docking site nonunion (nine cases), poor osteogenesis (five cases), severe chronic pain (five cases) and psychological problems (four cases) (Table 1).

**Table 1:** Demographic data of 23 patients.

Case No.	Age	Gender (M/F)	Open or closed (O/C)	Location	Medical diseases	Reasons for removing the external fixator
1	52	F	O	Femur	Hypertension	Poor osteogenesis
2	43	M	C	Tibia	None	Severe chronic pain
3	45	M	O	Femur	None	Docking site nonunion
4	38	F	O	Tibia	None	Poor osteogenesis
5	21	M	O	Tibia	None	Psychological problems
6	55	M	C	Tibia	Diabetes, Hypertension	Docking site nonunion
7	37	M	C	Femur	None	Psychological problems
8	34	M	O	Femur	None	Severe chronic pain
9	47	M	O	Tibia	Hypertension	Docking site nonunion
10	59	F	C	Tibia	Diabetes	Docking site nonunion
11	29	F	C	Tibia	None	Severe chronic pain
12	28	M	C	Femur	None	Severe chronic pain
13	36	F	O	Tibia	None	Docking site nonunion
14	27	M	O	Tibia	None	Docking site nonunion
15	43	M	O	Tibia	Hypertension	Poor osteogenesis
16	64	M	O	Tibia	Diabetes, Hypertension	Docking site nonunion
17	53	F	O	Femur	Nephropathy	Psychological problems
18	26	M	C	Tibia	None	Docking site nonunion
19	42	M	O	Femur	None	Psychological problems
20	33	F	C	Tibia	None	Poor osteogenesis
21	39	M	C	Tibia	None	Severe chronic pain
22	36	M	O	Tibia	None	Poor osteogenesis
23	55	F	O	Tibia	Hypertension, CHD	Docking site nonunion

CHD stands for coronary artery disease.

### Operation evaluation and complications

The mean residual bone defect length after removing the external fixator was (2.9 ± 1.9) cm. The mean time in external fixator was (9.5 ± 3.4) months. The average follow-up time was (23 ± 3) months. According to Paley score, the overall excellent and good rate of bone defect healing was 91.3% (excellent in 17 cases, good in four cases and fair in two cases). With respect to complications, two cases suffered from docking site nonunion again. They were treated by bone graft and augmented plate fixation. Six months later, the docking site healed well. Three cases had limb shortening (1.0 cm, 2.5 cm and 2.8 cm). Patients with 2.5-cm and 2.8-cm limb shortening had to wear thick insoles. No infection recurrence was found in these cases. There were no serious complications, such as nerve and vascular injuries or osteofascial compartment syndrome (Table 2).

**Table 2:** The operation evaluation and complications of 23 patients.

Case No.	Residual bone defects (cm)	Time in external fixator (month)	Description of operation	Complication	Paley score	Follow-up time (month)
1	7.7	15.0	IM + BG + AP	Nonunion	Excellent	22
2	3.9	7.6	IM + BG	None	Excellent	18
3	1.0	7.5	IM + BG	None	Excellent	25
4	5.3	4.2	IM + BG	Limb shortening	Fair	23
5	2.5	6.8	IM + BG	None	Excellent	22
6	1.0	8.9	IM + BG	None	Good	23
7	3.5	6.4	IM + BG	None	Excellent	19
8	2.8	6.8	IM + BG	None	Good	26
9	1.0	8.0	IM + BG	None	Excellent	23
10	1.0	10.4	IM + BG	None	Excellent	22
11	3.7	12.2	IM + BG	None	Excellent	20
12	3.0	7.4	IM + BG	Limb shortening	Fair	18
13	1.0	6.8	IM + BG	None	Excellent	19
14	1.0	14.7	IM + BG	None	Excellent	18
15	5.5	15.3	IM + BG + AP	Nonunion	Excellent	24
16	1.0	10.3	IM + BG	None	Good	19
17	3.6	6.6	IM + BG	None	Excellent	28
18	1.0	5.8	IM + BG	None	Excellent	27
19	2.6	6.7	IM + BG	None	Excellent	27
20	4.3	11.3	IM + BG	None	Excellent	24
21	3.5	13.4	IM + BG	None	Excellent	27
22	5.0	12.7	IM + BG	Limb shortening	Good	25
23	1.5	14.3	IM + BG	None	Excellent	20

IM stands for intramedullary nail. BG stands for bone graft. AP stands for auxiliary plate.

.

## Discussions

Traumatic large bone defects are a complex injury faced by trauma surgeons. Such patients are often secondary to infection after internal fixation or serious open fractures. The treatment cycle is long and the cost is high. Patients and their families face great economic burden and mental pressure.

So far, the treatment methods for large segmental bone defects include autologous bone transplantation, vascularized fibula transplantation, Masquelet technique, Ilizarov technique, etc. (2–9, 15, 16). Masquelet technique is essentially a bone transplant technique. Due to the limited autologous bone mass, this technique may not be applicable for large segmental bone defects. Bone transport represents one of the most useful Ilizarov techniques for large segmental bone defects. The basic principles of bone transport include stable external fixation, anatomical alignment, low-energy osteotomy and well controlled mechanical traction (17, 18). It has a high final success rate. Testa et al. reported a 96% good bone defect healing rate and an 84% good functional recovery rate in their study using bone transport technique (19). Tetsworth et al. compared the effects of bone transport with acute shortening/lengthening for the treatment of infected tibial nonunion. 95% patients receiving bone transport therapy achieved good radiological and functional bone healing results (20). However, some reasons may prevent its successful completion, such as docking site nonunion, poor osteogenesis, severe chronic pain or psychological problems.

Several scholars developed “accordion technique” to deal with docking site nonunion (21). That is, in order to promote healing, surgeons perform repeated distraction and compression at the docking site. Yet, due to the hardening of both bone defect ends and the occupation of local scar tissues, this technique sometimes could not solve the problem. Other scholars tried to trim the bone defect ends and perform autologous bone graft. In this method, patients need continuing to wear the transport fixator until docking site healing. This brings great inconvenience to patients’ life. For patients with poor osteogenesis of the transport segment, autologous bone graft can be performed directly. However, the transport frame is a relatively stable structure, and its biomechanical stability is worse than that of an intramedullary nail or a plate. This relatively stable structure is not conducive to the healing of bone graft. Moreover, the amount of autologous bones is limited. Yet, patients with poor osteogenesis often need a large amount of transplanted bones. The limited autologous bones may not meet the demands. Chronic pain exists in many patients who accept bone transport therapy. The causes of chronic pain include pin-tract infection, soft tissue irritation and cutting, tissue spasm, pin-tract loosening, etc. Most chronic pain was relieved after symptomatic treatment. However, as a variety of factors are intertwined, some patients show long-term chronic pain, which is unbearable. These patients insist on removing the external fixator and switching to other treatment methods. Besides, several patients wear the external frame for a long time, causing mental and psychological problems (10, 11). They urged removal of the external frame and the termination of treatment.

Several scholars used “bone transport over an intramedullary nail” technique to repair large bone defects and have achieved good results (22, 23). This technique can significantly reduce the time in frame and decrease the frame-related complications. However, due to the simultaneous use of internal and external fixation devices, patients are at great risk of infection recurrence. Lambiris et al. (24) found that intramedullary nailing during the consolidation phase after bone lengthening or bone transport was a treatment option for delayed callus maturation or docking site nonunion. It could reduce prolonged use of the external fixator. In the study of Emara et al. (25), when the transported segment reached the docking site, they offered the patients removal of the external fixator, and replacement by intramedullary fixation with bone graft at the docking site. It could achieve complete healing for infected nonunion of the tibial shaft with shorter duration of external fixation and give the same functional and bony outcome as the classic bone transport technique. Emara et al. drew a conclusion that it was a relatively safe technique but the risk of infection recurrence must be explained to the patients (26). The above scholars mainly used sequential nailing technique in patients with docking site nonunion. We have expanded the application of sequential nailing technique. In our study, 23 patients for different reasons of removing the external fixator were treated by sequential nailing technique.

Our modified technique has some advantages. First of all, it is superior to bone graft and plate fixation. As we know, patients with large segmental bone defects often have repeated operations and the local soft tissue conditions are poor. Plate fixation is difficult for these patients and the plate is easily exposed. This may cause infection recurrence. As the plate fixation is eccentric fixation, it is not conducive to early weight-bearing and functional exercises. On the contrary, intramedullary fixation is less affected by soft tissue conditions compared to plate fixation. It belongs to central fixation, which is reliable and is hard to break. Besides, the intramedullary nail occupies part of the space for bone graft, which can reduce the amount of autologous bones. This is helpful for patients with poor osteogenesis who need a large amount of autologous bones. In addition, based on our results, sequential nailing technique eliminated most chronic pain. Patients were satisfied to accept the treatment method. Three cases had limb shortening after operation in our study. After removal of the external fixator, the injured limb is prone to a certain degree of shortening due to the elastic retraction of soft tissues. Therefore, before sequential nailing, it is necessary to measure the length of the healthy side, take this as the standard, and pull the injured limb to restore the initial length as far as possible. In our study, two patients needed to wear thickened insoles because of limb shortening, but their satisfaction was still high. Another two cases encountered nonunion again after sequential nailing and bone graft. These two patients had partial bone resorption at the bone graft area. In order to reduce the occurrence of bone resorption and nonunion, the amount of autologous bones should be sufficient, mainly cancellous bones. The metabolic activity of cancellous bones was eight times higher than that of cortical bones (26). Although in our study there was no infection recurrence, we were still worried about this complication. Therefore, before replacing into an intramedullary nail and bone graft, the chief surgeon should ensure that there is no active infection. In addition, surgeons should ensure that the pin-tracts heal and the infection-related indexes are normal. If there is active or suspected infection, thorough debridement should be performed again. After the infection is completely controlled, sequential nailing could be carried out.

### Limitations

There were still some deficiencies in our research. The number of cases included in this study was small. The follow-up time was relatively short. This was a retrospective study without setting up a control group. The chief surgeon had a certain subjective bias when deciding when to remove the external fixator. Nevertheless, these factors did not affect the current conclusions. We will improve these situations in further research.

## Conclusions

Bone transport combined with sequential nailing technique could shorten the external fixation time, overcome the inconvenience of the external frame to patients, eliminate chronic pain and be easy for patients to accept. Patients using this modified technique achieved high satisfaction. So it should be further widely used.

## Data Availability

The original contributions presented in the study are included in the article/Supplementary Material, further inquiries can be directed to the corresponding author.
